# Supplementary material for: Galactose in the Post-Weaning Diet Programs Improved Circulating Adiponectin Concentrations and Skeletal Muscle Insulin Signaling
Source: Int J Mol Sci. 2022 Sep 6;23(18):10207. doi: 10.3390/ijms231810207 (PMC9499164; doi:10.3390/ijms231810207)
Supplement: Supplementary file 1 [file ijms-23-10207-s001.zip › ijms-1849842-supplementary.pdf]

Supplemental materials

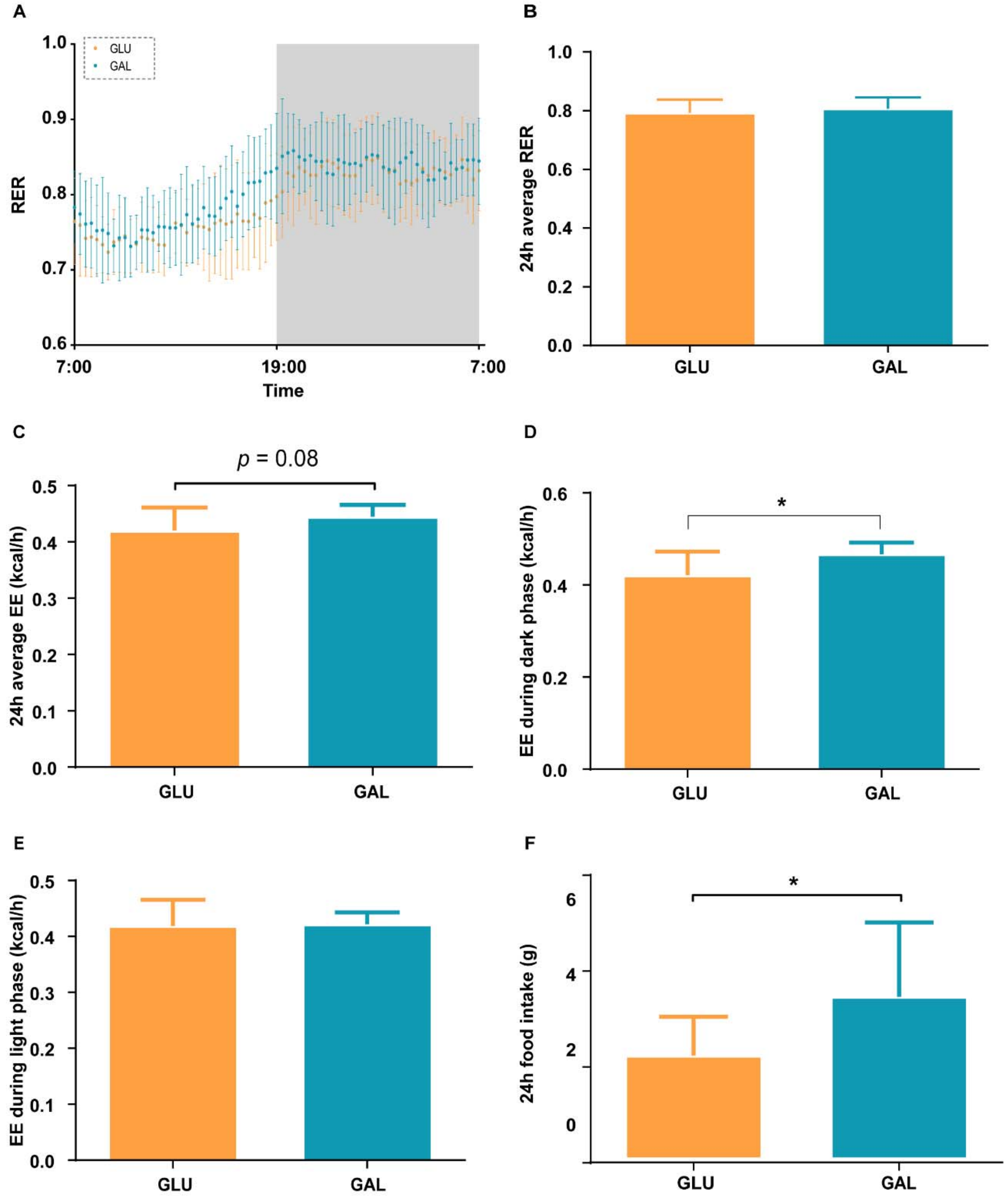

Figure S1: Indirect calorimetry (Inca) measurements in GLU and GAL mice after 7-week on HFD (postnatal day 93-day 98). A) Respiratory exchange ratio (RER) patterns, B) 24-hour average RER, C) average energy expenditure (EE) during 24-hour Inca measurements. D) average EE during dark phase. E) average EE during light phase. F) HFD food intake during the 24-hour Inca measurements, Values are expressed as mean ± SD, n = 12 -14, * p<0.05.

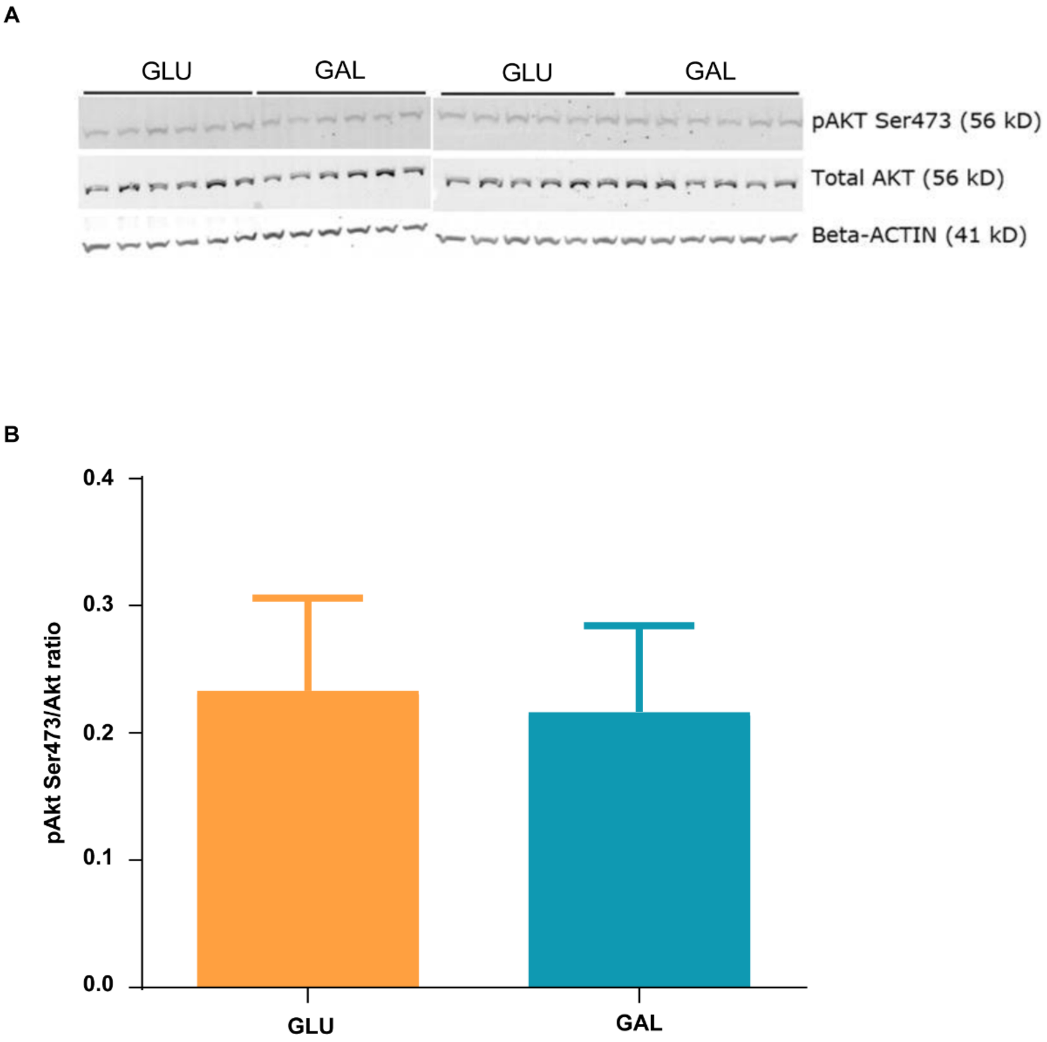

Figure S2. Western blot of AKT activation in gWAT of GLU and GAL mice, PN105, 15 minutes after 2.0 mg/kg BW glucose bolus. A) Overview of blot results of activated pAKT-Ser473, total AKT, and β-actin (loading control), B) Quantification of pAKT-Ser473 / total AKT ratio, corrected for β-actin levels, as marker of cellular insulin signalling. Values are given as mean ± SD, *n*=12.

Table S1: Diet compositions

	Glucose diet		Galactose diet		High fat diet	
	Mass (g)	Energy (kcal)	Mass (g)	Energy (kcal)	Mass (g)	Energy (kcal)
**Casein**	200.0	800	200.0	800	233.0	932
**L-cysteine**	3.0	12	3.0	12	4.0	16
**Wheat starch**	278.0	1112	278.0	1112	300.0	1,200
**Sucrose**	0.0	0	0.0	0	155.4	622
**Glucose**	322.0	1,288	161.0	644	0.0	0
**Fructose**	29.5	118	29.5	118	0.0	0
**Galactose**	0.0	0	161.0	644	0.0	0
**Cocos oil**	12.6	113	12.6	113	0.0	0
**Sunflower oil**	49.0	441	49.0	441	0.0	0
**Flaxseed oil**	8.4	76	8.4	76	4.0	36
**Palm oil**	0.0	0	0.0	0	206.0	1,854
**Cholesterol**	0.030	0	0.030	0	0.097	0
**Cellulose**	50.0	0	50.0	0	50.0	0
**Mineral mix**	35.0	31	35.0	31	35.0	31
**Vitamin mix**	10.0	39	10.0	39	10.0	39
**Choline bitartrate**	2.5	0	2.5	0	2.5	0
**Total energy (kcal/kg)**		4,030		4,030		4,730
**Protein (en%)**		20		20		20
**Carb (en%)**		64		64		40
**Fat (en%)**		16		16		40

Mass is given in gram/kilogram.

Table S2: Primer sequences and annealing temperatures in RT-qPCR

Tissue	Genes	Forward Primer (F, 5'-3')<br>Reverse Primer (R, 5'-3')	Annealing Temperature (°C)
**Gonadal WAT**	*Irs1*	F: TTAGGCAGCAATGAGGGCAA<br>R: TCTTCATTCTGCTGTGATGTCCA	60
	Irs2	F: GCACCTATGCAAGCATCGAC<br>R: GCGCTTCACTCTTTCACGAC	60
	Tbc1d4 (As160)	F: CTGGAAGCAAGAAGAGATGAGC<br>R: ATTTCCCCTCGCCGACTTTT	62
	AdipoQ	F: GCCACTTTCTCCTCATTTCTGTCTG<br>R: CATCTCCTTTCTCTCCCTTCTCTCC	60
	Atgl	F: ACCACCCTTTCCAACATGCTACC<br>R: GCTACCCGTCTGCTCTTTCATCC	58
	Cpt1a	F: AAAGATCAATCGGACCCTAGACA<br>R: CAGCGAGTAGCGCATAGTCA	57
	Lpl	F: GGACTGAGAATGGCAAGCAACAC<br>R: GCAGTTCTCCGATGTCCACCTC	60
	Mrc1	F: GTTATGAAAGGCAAGGATGGATAC<br>R: TCAGTGAAGGTGGATAGAGTGG	58
	Saa3	F: AAAGAAGCTGGTCAAGGGTC<br>R: TGTCCCGTGAACTTCTGAAC	58
	S100a8	F: ACTTCGAGGAGTTCCTTGCG<br>R: TGCTACTCCTTGTGGCTGTC	58
	Mest	F: GATTCGCAACAATGACGGCA<br>R: ATCCAGAATCGACACTGTGG	57
	Rarres2	F: AAGCTCCAGCAGACCAACTG<br>R: TTTCCGCCTTCTCCCGTTTG	58
	B2m (ref)	F: CCCCACTGAGACTGATACATACGC<br>R: AGAAACTGGATTTGTAATTAAGCAGGTTC	60
	Canx (ref)	F: GCAGCGACCTATGATTGACAACC<br>R: GCTCCAAACCAATAGCACTGAAAGG	60
	Rps15 (ref)	F: CGGAGATGGTGGGTAGCATGG<br>R: ACGGGTTTGTAGGTGATGGAGAAC	60
Liver	*AdipoR1*	F: CATCTACCTCTCCATCGTCTGTGTC<br>R: TGGGTACAACACCACTCAAGCC	60
	AdipoR2	F: CGCTTCTTTCCTGGCAAATGTG<br>R: CCCTGGTTCCTGGAGAGTATCAC	60
	Irf1	F: AGCATCTCGGGCATCTTTCG<br>R: GAGTGATTGGCATGGTGGCTTT	60
	Stat1	F: GGATCGCTTGCCCAACTCTT<br>R: CACTGTGACATCCTTGGGCT	60
	S100a8	F: ACTTCGAGGAGTTCCTTGCG<br>R: TGCTACTCCTTGTGGCTGTC	60
	Saa3	F: AAAGAAGCTGGTCAAGGGTC<br>R: TGTCCCGTGAACTTCTGAAC	58
	Screbf1	F: TCCAGTGGCAAAGGAGGCAC<br>R: CAGCATGCTCATTCGCTGCC	60
	Acox1	F: TGCGGTGGGCACGGCTATTC<br>R: CGCTGGCTCGGCAGGTCATT	60
EDL muscle	*AdipoR1*	See above	60
	AdipoR2	See above	60
	Rarres2	See above	58
	Irs1	See above	60
	Irs2	See above	60
	PDK4	F: TCAGTGACTCAAAGACGGGAAACC<br>R: TGTGGTGAAGGTGTGAAGGAACG	60
	Cpt1b	F: ATCGCCGCAAACTGGACCGT<br>R: ACCCAGTGCCATGACCGGCT	60
	Acly	F: TGGGCTTCATTGGGCACTACC<br>R: AGGGCTCCTGGCTCAGTTACA	62
	Acacb	F: TCTTCACGTTCAGAGCGAGGGAT<br>R: ATCTTGTGGTTGGCACAGGGCA	56
	Pgc1a	F: CCCTGCCATTGTTAAGACC<br>R: TGCTGCTGTTCCTGTTTTC	60

Reference genes are given in the segment of gWAT, but were used for all three tissues.
